# Evolution of Multitarget Strategies for Alzheimer’s Disease: From Cholinergic Inhibition to Network-Oriented Therapeutic Design (2006–2025)

**DOI:** 10.3390/ph19071024

**Published:** 2026-06-30

**Authors:** Jaime Mella, Alejandro Vega-Muñoz, Mauricio Soto, Daniel Moraga, Javier Campanini-Salinas, Eduardo Sandoval-Obando, Nicolás Contreras-Barraza, Guido Salazar-Sepúlveda, Natalia Salas-Guzmán, Remik Carabantes-Silva, Marco Mellado

**Affiliations:** 1 Instituto de Química, Facultad de Ciencias, Universidad de Valparaíso, Valparaíso 2360102, Chile; jaime.mella@uv.cl; 2 Centro de Investigación, Desarrollo e Innovación de Productos Bioactivos (CInBIO), Universidad de Valparaíso, Valparaíso 2360102, Chile; 3 Laboratorio de Bienestar y Comportamiento Organizacional, Facultad de Medicina y Ciencias de la Salud, Universidad Central de Chile, Santiago 8330507, Chile; alejandro.vega@ucentral.cl; 4 Centro de Investigación en Medicina de Altura, Universidad Arturo Prat, Santiago 8340232, Chile; 5 Departamento de Química, Universidad Técnica Federico Santa María, Av. España 1680, Valparaíso 2340000, Chile; mauricio.sotoc@usm.cl; 6 Laboratorio de Fisiología, Departamento de Ciencias Biomédicas, Facultad de Medicina, Universidad de Tarapacá, Arica 1000000, Chile; dmoraga@academicos.uta.cl; 7 Escuela de Química y Farmacia, Facultad de Ciencias, Universidad San Sebastián, Puerto Montt 5501842, Chile; javier.campanini@uss.cl; 8 Escuela de Psicología, Facultad de Ciencias Sociales y Humanidades, Universidad Autónoma de Chile, Temuco 4800916, Chile; eduardo.sandoval@uautonoma.cl; 9 Facultad de Ciencias Económicas y Administrativas, Pontificia Universidad Católica de Valparaíso, Valparaíso 2340025, Chile; nicolas.contreras@pucv.cl; 10 Facultad de Ingeniería, Universidad Católica de la Santísima Concepción, Concepción 4090541, Chile; gsalazar@ucsc.cl; 11 Facultad de Ingeniería y Negocios, Universidad de Las Américas, Concepción 4090940, Chile; 12 Facultad de Educación y Ciencias Sociales, Universidad Finis Terrae, Santiago 7501015, Chile; nsalas@uft.cl; 13 Facultad de Ciencias Naturales, Matemáticas y del Medio Ambiente, Universidad Tecnológica Metropolitana, Santiago 8330383, Chile; r.carabantes@utem.cl; 14 Dirección de Investigación, Universidad Bernardo O’Higgins, Santiago 8370993, Chile; 15 Laboratorio de Espectroscopía y Química Aplicada, Grupo de Investigación en Ciencias Biomédicas Aplicadas, Universidad Central de Chile, Santiago 8330546, Chile

**Keywords:** Alzheimer’s disease, multitarget-directed ligands (MTDLs), network pharmacology, drug discovery, β-amyloid aggregation, oxidative stress, blood–brain barrier

## Abstract

**Background:** Alzheimer’s disease (AD) is a complex neurodegenerative disorder and a major global health challenge. The traditional “one drug–one target” paradigm has shown limitations in addressing its multifactorial nature. Multitarget-directed ligands (MTDLs), designed to modulate multiple pathological pathways, have emerged as a promising therapeutic strategy. **Objectives:** To examine the structural, thematic, and temporal evolution of multitarget strategies for AD treatment between 2006 and 2025. **Methods:** A total of 1184 Web of Science-indexed articles were analyzed. Publication growth, h-index, author productivity, institutional and national contributions, and keyword co-occurrence networks were evaluated using VOSviewer. Bibliometric laws (Price, Bradford, Zipf, and Lotka) were applied to characterize productivity patterns and thematic organization. **Results:** Multitarget research shows exponential growth, suggesting a consolidation of the MTDL paradigm. China, India, the United States, Italy, and Spain were the most productive countries. Early studies focused on cholinesterase inhibition, particularly acetylcholinesterase-based hybrids. The field expanded to include β-amyloid aggregation, oxidative stress, metal chelation, and blood–brain barrier permeability. Recent trends emphasize integration of computational approaches, including molecular docking, molecular dynamics, virtual screening, and network pharmacology, alongside targets such as BACE1 and GSK-3β. **Conclusions:** Multitarget strategies have evolved toward a systems-oriented framework. Despite advances, challenges remain in reducing cholinesterase dependency and improving translational validation. This study provides a framework to interpret therapeutic evolution and guide future network-based drug design.

## 1. Introduction

Alzheimer’s disease (AD) is a complex neurodegenerative disorder characterized by the dynamic interplay among multiple pathophysiological processes, including β-amyloid (Aβ) aggregation, hyperphosphorylation of the tau protein, oxidative stress, neuroinflammation, and mitochondrial dysfunction. Although the amyloid hypothesis has historically dominated the conceptual framework of the disease, recent evidence suggests that neurodegenerative progression responds to a broader interconnected network of cellular and molecular events [1,2]. Several studies have highlighted the critical role of redox imbalance as a convergent node in AD pathogenesis, linking mitochondrial dysfunction with progressive synaptic damage [3,4]. Complementarily, sustained microglial activation emerges as a relevant modulator of neuronal deterioration [5], while emerging mechanisms such as ferroptosis have been proposed as novel pathways of cell death involved in disease progression [6]. In parallel, disruption of the blood–brain barrier and alteration of the neurovascular axis have expanded the traditional understanding previously centered exclusively on protein deposition [7]. This conceptual shift suggests a possible transition from linear models toward systemic frameworks based on interdependent biological networks. Beyond this network-level perspective, AD progression is also shaped by synaptic dysfunction, circuit-level disconnection, tau-mediated neurotoxicity, neuronal network failure, and impaired plasticity, which are central to cognitive decline and remain relevant therapeutic nodes for multitarget intervention.

The growing multifactorial understanding of AD has driven the development of multi-target pharmacological strategies (Multi-Target Directed Ligands, MTDLs), designed to simultaneously modulate multiple interconnected pathological nodes. Unlike the classical “one drug–one target” paradigm, the MTDL approach seeks to intervene coordinately in processes such as the inhibition of acetylcholinesterase (AChE) and butyrylcholinesterase (BuChE), Aβ aggregation, tau hyperphosphorylation, oxidative stress, metal dyshomeostasis, and neuroinflammation [8,9].

In its early stages, multi-target design was structured around the molecular hybridization of classical cholinergic inhibitors, such as tacrine or donepezil, with antioxidant or metal-chelating fragments, generating compounds with symptomatic activity and potential disease-modifying properties [10,11]. Subsequently, the paradigm evolved toward more sophisticated architectures integrating dual enzymatic inhibition (AChE/BuChE, AChE/BACE1, AChE/MAO), anti-aggregating properties against Aβ, and neuroprotective capacity associated with redox modulation [12,13]. More recently, the incorporation of molecular docking, molecular dynamics, virtual screening, and network pharmacology has enabled a more precise structural rationalization of the desired polypharmacology [14,15], facilitating the identification of compounds with balanced multitarget profiles and optimized pharmacokinetic properties, including blood–brain barrier permeability. Likewise, the integration of artificial intelligence and machine learning is beginning to redefine rational drug discovery in this context [16,17,18].

Nevertheless, despite conceptual and methodological advances, significant challenges remain, particularly the translational gap evidenced by the predominance of preclinical studies, where most of the reported MTDL compounds are currently found, thereby limiting their robust clinical validation.

In this context, the present study conducts a comprehensive bibliometric analysis of research on the development of multi-target strategies for the potential treatment of Alzheimer’s disease indexed in the Web of Science database. Based on a structured corpus of 1184 articles, classical bibliometric laws (Lotka, Bradford, and Price) are integrated with scientific mapping tools (VOSviewer 1.6.20) to characterize publication dynamics, influential actors, and the thematic evolution of the field. This approach provides a quantitative and reproducible framework to contextualize chemical and biological efforts, identify emerging directions, and contribute to the structural understanding of the maturation of the multi-target paradigm.

## 2. Results and Discussion

Using the Web of Science Core Collection as the data source, a systematic bibliometric analysis was conducted through the thematic query TS = (((Dual NEAR/0 Target) OR (Multi NEAR/0 Target)) AND (Alzheimer)), which enabled the retrieval of records explicitly containing the expressions “dual target,” “multi target,” and “Alzheimer” in the title, abstract, or keywords. This initial search strategy identified a total of 1685 documents. By restricting the results exclusively to research articles, the dataset was reduced to 1205 records. Subsequently, after applying a temporal filter covering the period between 2006 and 2025, the final corpus consisted of 1184 documents.

From a conceptual perspective, the retrieved literature is mainly focused on the development of bioactive compounds with two or more pharmacological functions integrated within a single chemical entity. This multi-target approach is widely regarded as a relevant strategy for the treatment of complex diseases such as Alzheimer’s disease, as it enables the simultaneous modulation of multiple therapeutic targets and may contribute to reducing polypharmacy [19].

It is important to note that bibliometric analyses are inherently descriptive and do not allow for causal inference. Therefore, interpretations regarding underlying drivers of scientific productivity (e.g., funding, epidemiology, or policy) should be considered as plausible contextual explanations rather than demonstrated causal relationships.

Continuous and sustained scientific production associated with multitarget strategies for Alzheimer’s disease is observed from 2006 onward, which justifies the selection of the analyzed temporal interval. This pattern is consistent with Price’s law, which describes the exponential growth of scientific literature during the consolidation phases of a research field. From 2006 onward, a progressive and stable increase in the number of publications becomes evident, suggesting the transition toward a phase of structured development in the field. The temporal evolution of scientific production reveals a sustained increase in the number of publications over the period analyzed. According to the bibliometric data, this trend reflects the transition from an emerging research topic toward a more consolidated and expanding scientific area. The progressive growth observed may be associated with increasing scientific interest in multitarget strategies as a response to the biological complexity of Alzheimer’s disease.

### 2.1. Growing Understanding in the Research on Multi-Target Strategies for the Treatment of Alzheimer’s Disease

**Figure 1:**
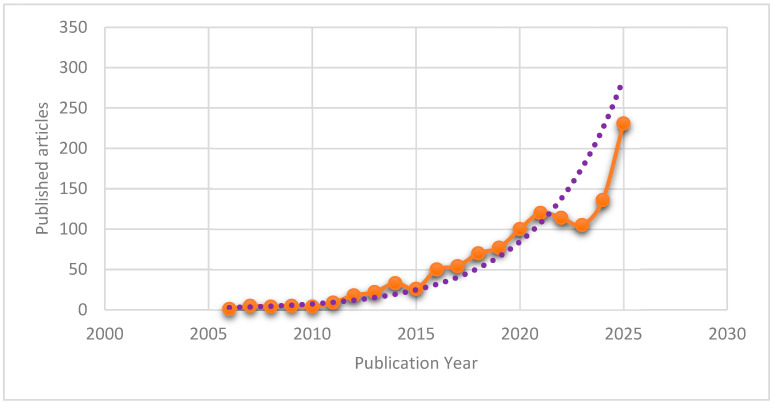
Temporal distribution of publications per year from 2006 to 2025 on the study of multi-target compounds as a therapeutic strategy for the treatment of Alzheimer’s disease. Solid orange line: data on the number of items retrieved from the database. Dashed purple line: fitting of the data to an exponential function curve.

From 2006 onward, the scientific production associated with this topic exhibits a sustained growth trend, which can be adequately described by an exponential model fitted according to the equation y = 4 × 10^−214^ e^0.2454x^, with x equal to publication year (2006 to 2025) and a high coefficient of determination (r^2^ = 0.9266), as shown in Figure 1. This high degree of fit indicates that most of the variability observed in the annual number of publications is explained by an exponential growth pattern, characteristic of research fields undergoing active expansion.

This temporal behavior is consistent with Price’s law, which postulates that scientific production in emerging or consolidating areas tends to grow exponentially until reaching a critical mass of knowledge [20]. In this context, the observed growth may be associated with the progressive consolidation of the multi-target approach as a relevant paradigm in chemical and pharmacological sciences, aimed at the development of chemical entities capable of simultaneously modulating two or more pharmacological targets, particularly in the therapeutic management of complex diseases such as Alzheimer’s disease.

Considering the totality of the analyzed records, it is observed that half of the corpus corresponds to 592 documents. Within this subset, the most recent fraction (contemporary articles) is concentrated in the period 2022–2025, reaching a total of 586 publications. Although a substantial proportion of research on the development of multi-target compounds for the treatment of Alzheimer’s disease is situated within this time interval, Figure 1 reveals a deceleration in the annual growth rate of publications. This behavior may be interpreted as the result of multiple concurrent factors. On the one hand, the high degree of experimental complexity inherent to this field (e.g., extensive synthesis, characterization, and biological validation) may slow the cycles of scientific production. On the other hand, operational limitations associated with the COVID-19 pandemic, particularly those related to access to experimental infrastructure and researcher mobility, may have contributed to delays in data generation and the completion of ongoing studies. Taken together, these elements suggest that the field may be transitioning from a phase of accelerated growth toward a scenario of progressive consolidation, characterized by more selective and methodologically robust scientific production.

### 2.2. Collaboration Networks in the Research on Multi-Target Strategies for the Treatment of Alzheimer’s Disease

**Figure 2:**
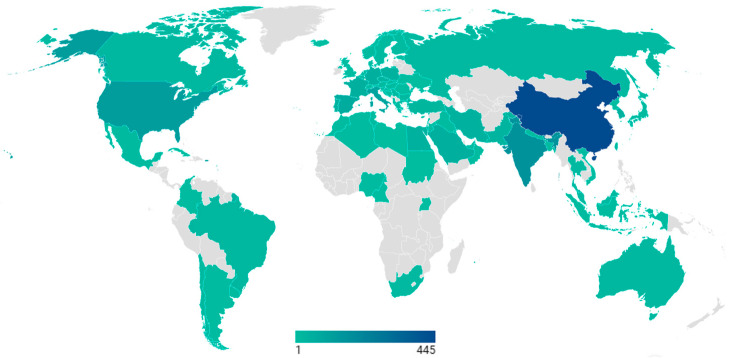
Countries that have contributed to the study of multi-target compounds as a therapeutic strategy for the treatment of Alzheimer’s disease. The color gray indicates a lack of scientific output in the field under study.

During the analyzed period (2006–2025), global scientific production related to the design and development of multi-target molecules capable of acting on two or more complementary therapeutic targets for the potential treatment of Alzheimer’s disease shows a marked geographical concentration. The analysis of contributions by country was conducted using a full-counting approach, in which each country associated with an article receives full credit, regardless of the number of co-authors or affiliations involved. Under this criterion, 1742 affiliation records were identified, consistent with the high intensity of international collaboration in this field of research. In this context, five countries emerge as the principal contributors to this line of research: China (445 records, 26%), India (162 records, 9%), the United States of America (123 records, 7%), Italy (96 records, 6%), and Spain (66 records, 4%), which together account for 52% of the contributions, as illustrated in Figure 2.

In the case of China, although Alzheimer’s disease and other dementias represent a relevant fraction of the national epidemiological profile, several reports suggest that its scientific leadership is primarily driven by structural factors within the research system rather than by the epidemiological burden alone [21]. In particular, the central role of the National Natural Science Foundation of China (NSFC), together with the sustained increase in research and development expenditure (from 1.31% of GDP in 2005 to 2.56% in 2022), has enabled the consolidation of a highly productive scientific infrastructure in the biomedical and pharmacological fields [22,23].

India, for its part, exhibits high productivity despite the absence of consolidated epidemiological records on Alzheimer’s disease in the WHO databases [24]. This pattern suggests that scientific production in this country is mainly driven by institutional strengthening and the diversification of public funding agencies [22], such as the University Grants Commission, the Department of Science and Technology, and the Council of Scientific and Industrial Research, as well as by the presence of organizations specialized in brain aging and neurodegeneration [25].

In contrast, the scientific productivity of the United States of America appears to be supported both by a high epidemiological burden of the disease, ranked as the second leading cause of death, and by a robust investment in research and development, which increased from 1.95% of GDP in 2005 to 2.67% in 2022 [22,26]. In this context, federal funding, primarily channeled through the National Institutes of Health (NIH) and the National Institute on Aging (NIA), plays a strategic role in promoting research aimed at the development of new therapeutic strategies.

Finally, Italy and Spain exhibit comparable levels of productivity, characterized by strong integration within European research networks. In both cases, the combination of national funding, European Union programs, and cooperation schemes such as COST, together with a significant epidemiological burden of neurodegenerative diseases [27,28], has enabled the maintenance of relevant scientific production, even though their levels of investment in R&D remain lower than those of the leading countries [22].

Taken together, these results indicate that scientific productivity in the development of multi-target compounds for Alzheimer’s disease is primarily determined by the structural capacity of national science and technology systems, access to competitive funding, and integration into international collaboration networks, rather than by the epidemiological burden of the disease considered in isolation.

### 2.3. Leading Institutions in the Research on Multi-Target Strategies for the Treatment of Alzheimer’s Disease

During the analyzed period (2006–2025), a total of 1668 institutions worldwide were identified with at least one affiliation in scientific articles related to the development of multi-target compounds for Alzheimer’s disease. The analysis of institutional distribution reveals a widely dispersed scientific production, without a dominant concentration in a small number of academic or research entities. In this context, Sun Yat-sen University emerges as the institution with the highest individual contribution, accounting for 0.8% of the total publications, while the 100 most productive institutions collectively represent approximately one-third of the total scientific production. Considering this scenario, Table 1 presents the ten institutions with participation exceeding 0.5% of the total publications in this research area.

When comparing the most prolific institutions in the development of multi-target compounds for Alzheimer’s disease with the countries that concentrate the highest scientific production in this area, it is observed that six of the ten identified institutions belong to these leading countries, particularly China, Italy, and Spain. In the case of China, Sun Yat-sen University, China Pharmaceutical University, Nanyang Normal University, and the Chinese Academy of Sciences stand out, which is consistent with the sustained leadership of this country at the national level. In Europe, the Università degli Studi di Bari Aldo Moro (Italy) and the Consejo Superior de Investigaciones Científicas (CSIC, Spain) are particularly prominent, suggesting a possible link with the strength of consolidated research systems on the continent.

Complementarily, Table 1 also includes institutions belonging to countries that do not rank among the most prolific at the global level, such as Jagiellonian University (Poland) and the Universities of Lisbon and Coimbra (Portugal). The presence of these institutions could be related, in part, to their integration into international collaboration networks, particularly through co-authorship links with the CSIC. Likewise, the University Hospital Hradec Králové (Czech Republic) shows indirect connections with the CSIC mediated by its collaboration with Jagiellonian University. These relationships, visualized in Figure S1, highlight the role of certain institutions as articulating nodes within the research network, facilitating the participation of entities from countries with lower aggregate production in the development of multi-target strategies for Alzheimer’s disease.

The comparative analysis of the scientific production of the different institutions reveals a convergent evolution of the multi-target paradigm in Alzheimer’s disease research, from early approaches centered on classical enzymatic targets toward more integrated, systemic, and translational strategies.

In institutions such as Sun Yat-sen University and the Chinese Academy of Sciences, a transition is observed from first-generation multi-target ligands, based on the simultaneous inhibition of cholinesterases and modulation of the amyloid cascade, toward more complex approaches incorporating network pharmacology, regulation of intracellular signaling pathways, and control of neuroinflammatory processes [29,30,31,32]. This shift may be associated with a more holistic understanding of Alzheimer’s pathophysiology, moving beyond the perspective centered exclusively on the amyloid hypothesis.

China Pharmaceutical University and Nanyang Normal University demonstrate conceptual continuity within the multitarget approach, accompanied by functional and methodological expansion. While earlier works align with the classical design of MTDLs through pharmacophoric hybridization [33,34] more recent studies extend the scope toward dual diagnostic probes and the use of artificial intelligence for the design and screening of multitarget libraries, integrating in vivo validation and advanced translational models [35,36].

Within the European context, the Università degli Studi di Bari Aldo Moro, the University Hospital Hradec Králové, and Jagiellonian University show an evolution from classical cholinergic strategies toward second-generation multitarget approaches that incorporate neuroinflammation, protein aggregation (Aβ and tau), pro-inflammatory kinases, and neurotransmitter systems involved in the cognitive and behavioral symptoms of Alzheimer’s disease [32,37,38,39,40,41]. These developments could be related to the maturation of the concept of multi-targeting, oriented not only toward molecular efficacy but also toward clinical and functional relevance.

The CSIC and the Universidade de Lisboa present particularly coherent and complementary trajectories. From pioneering studies on the stereochemical optimization of multitarget ligands [42], the CSIC has evolved toward efficient multicomponent synthesis strategies for the rapid generation of multifunctional libraries [43]. In parallel, the Universidade de Lisboa has progressed from bifunctional antioxidant/anticholinesterase ligands inspired by natural products [44] toward more complex chemical entities capable of modulating cholinesterases, oxidative stress, and amyloid aggregation, strengthening this evolution through strategic international collaborations with the CSIC [45].

Finally, the University of Coimbra clearly illustrates the progression of the multitarget paradigm. Its initial work, developed in collaboration with the Universidade de Lisboa, falls within the foundational framework of bifunctional ligands directed toward oxidative stress and cholinergic dysfunction [43]. The most recent study demonstrates conceptual and methodological sophistication, orienting the design toward dual inhibitors of BACE1 and BuChE, integrating targets relevant in both early and advanced stages of Alzheimer’s disease, together with structure-based design, docking, cellular evaluation, and sustainable chemistry approaches [44].

Taken together, this body of evidence demonstrates a global evolution from multitarget approaches centered on classical enzymatic inhibition toward more integrated strategies, in which multi-targeting is redefined as the coordinated modulation of biological networks, inflammatory processes, protein aggregation, and neuropsychological functions, in accordance with the multifactorial complexity of Alzheimer’s disease.

### 2.4. Prolific Authors and Co-Authorship in the Research on Multi-Target Strategies for Treatment of Alzheimer’s Disease

**Figure 3:**
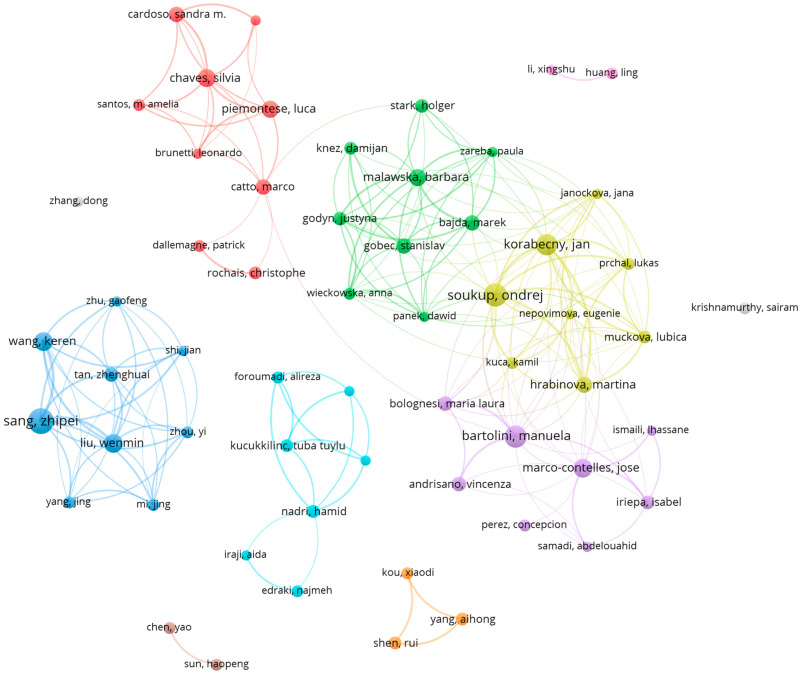
Co-authorship and collaborative networks in the development of multitarget strategies for Alzheimer’s Disease treatment. The colors correspond to the different clusters grouped by the VOSviewer program.

From a total of 1184 analyzed articles, the participation of 6833 researchers was identified, highlighting the breadth and diversity of the scientific community involved in the development of multi-target strategies for the treatment of Alzheimer’s disease. In order to identify the most prolific authors, Lotka’s law [46,47,48] was applied, estimating the productivity threshold as the square root of the total number of authors (~82.66). Based on this criterion, 59 authors concentrating the highest scientific production in this field were selected. When classifying these authors according to their level of productivity, it is observed that four researchers exhibit a sustained rate of one or more contributions per year throughout the analyzed period. Zhipei Sang (Nanyang Normal University), Ondrej Soukup (University Hospital Hradec Králové), Manuela Bartolini (University of Bologna), and Jan Korabecny (University of Defense) emerge as the most active authors, with 20 or more publications related to multi-target strategies in Alzheimer’s disease. When contrasting the institutional affiliation of these authors with the results presented in Table 1, it becomes evident that only two of the four are linked to the most prolific institutions (Nanyang Normal University and University Hospital Hradec Králové). This result suggests that high individual productivity does not depend exclusively on institutional volume but may instead be associated with internal collaboration dynamics and the consolidation of highly specialized research groups, a phenomenon that is clearly consistent with the co-authorship networks shown in Figure 3.

As shown in Figure 3, collaboration networks are organized into well-defined clusters, most of which exhibit a closed structure, with limited interactions between groups. This pattern is particularly evident in the blue cluster, led by Zhipei Sang (Nanyang Normal University), which includes nine co-authors and shows low external connectivity. A similar configuration is identified in the cyan (Hamid Nadri, University of Tehran), orange (Rui Shen, Tianjin University of Traditional Chinese Medicine), and pink (Huang Ling, Hainan University) clusters, which operate as relatively autonomous scientific communities. However, this trend is partially mitigated in the red cluster, where the co-authorship of Dr. Marco Catto (Università degli Studi di Bari Aldo Moro) with Dr. Holger Stark (Heinrich Heine University Düsseldorf), belonging to the green cluster, and with Dr. Maria Laura Bolognesi (Alma Mater Studiorum—University of Bologna), from the purple cluster, introduces intercluster links that facilitate the circulation of knowledge. A similar behavior, although with a higher degree of interconnection, is observed between the yellow and green clusters, suggesting the existence of more open and collaborative subnetworks. Taken together, these patterns indicate that, despite the predominance of closed communities, certain authors act as strategic linking nodes, facilitating the partial integration of collaboration networks in this research field.

The analysis of the distribution of collaboration clusters reveals that Dr. Zhipei Sang (Nanyang Normal University) is the most prolific author in the dataset and leader of the blue cluster, with 26 contributions focused on the development of multi-target strategies for Alzheimer’s disease. His early work focused on the hybridization of cholinesterase inhibitors with cinnamic acids, combining enzymatic and antioxidant activity [34]. In more recent stages, this research line has expanded through the integration of multiple pharmacophores within a single chemical entity and the exploration of anti-inflammatory effects, which is consistent with a progressive sophistication of the multitarget approach [49,50].

Dr. Ondrej Soukup (University Hospital Hradec Králové), the second most prolific author and leader of the yellow cluster, has 23 publications. His initial contributions were based on tacrine derivatives as cholinesterase inhibitors, supported by computational studies to identify key interactions [38]. Subsequently, his research evolved toward the incorporation of NMDA receptor agonist activity and the evaluation of blood–brain barrier permeability, broadening the pharmacological profile of the compounds [51].

Within the purple cluster, Dr. Manuela Bartolini (University of Bologna) stands out with 22 published articles. Her research trajectory began with the development of quinone derivatives with inhibitory activity against cholinesterases and Aβ aggregation [52]. In more recent work, she has maintained this structural platform while incorporating the evaluation of antioxidant activity in neuronal cell models, reinforcing the multifunctional nature of her proposals [53].

The red cluster is led by Dr. Silvia Chaves (Universidade de Lisboa), with 16 publications. Her initial studies focused on the combination of 3-hydroxy-4-pyridinone and benzofuran, generating hybrids with inhibitory activity against cholinesterases, metal chelation capacity, inhibition of Aβ aggregation, and antioxidant activity [54]. In more recent research, she has maintained these pharmacological targets while exploring new scaffold structures, such as rivastigmine [55].

In the green cluster, Dr. Barbara Malawska (Jagiellonian University) leads with 15 articles. Her early research employed isoindoline-1,3-dione derivatives as inhibitors of cholinesterases and Aβ1–42 aggregation, incorporating neuroprotective effects [40]. Subsequently, her approach expanded toward modulation of the GABAergic system, including studies in murine models, demonstrating a progression toward more translational approaches [56].

The cyan cluster is headed by Dr. Hamid Nadri (University of Tehran), with 11 publications. His early work employed aurones and donepezil-mimetic fragments, identifying key interactions through molecular docking [57]. In more recent studies, he explores coumarin derivatives with inhibitory activity against cholinesterases, Aβ1–42 aggregation, and neuroprotective effects against oxidative stress [58].

Finally, the orange cluster, led by Dr. Rui Shen (Tianjin University of Traditional Chinese Medicine) with 10 articles, shows a coherent research line based on xanthone and anthraquinone derivatives as cholinesterase inhibitors, metal chelators, and antioxidant agents, maintaining similar pharmacological targets over time [59,60].

**Figure 4:**
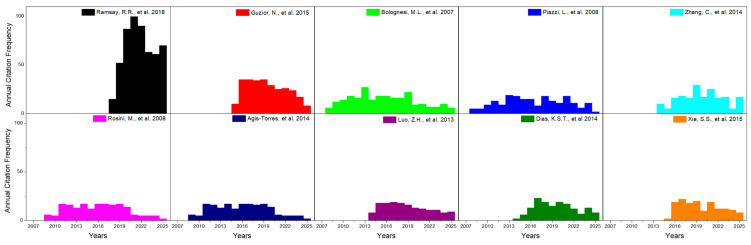
Annual citation distribution patterns of the ten most highly cited articles related to multitarget strategies for Alzheimer’s disease during the analyzed period (2006–2025). The y-axis represents yearly citation frequency rather than cumulative citation counts [8,9,10,11,12,13,14,15,61,62].

In the present study, the h-index of the analyzed dataset, comprising articles published between 2006 and 2025, was calculated, yielding a value of h = 71; that is, 71 articles with at least 71 citations each, with a citation range between 547 and 72 citations according to the Web of Science database. These values correspond to cumulative citation counts accumulated throughout the analyzed period rather than annual publication frequencies. When comparing this result with the scientific production growth trends described in Section 2.1, it is observed that most of the highest-impact articles are concentrated in the first half of the analyzed period (2006–2021), suggesting that the foundational works of the multitarget approach have played a decisive role in consolidating the field. Among the most influential articles are those by Ramsay et al. (2018) [8], Guzior et al. (2015) [9], Bolognesi et al. (2007) [10], Piazzi et al. (2008) [11], Zhang et al. (2014) [12], Rosini et al. (2008) [13], Agis-Torres et al. (2014) [14], Luo et al. (2013) [15], Dias et al. (2014) [61], and Xie et al. (2015) [62], presented in descending order according to the number of citations within the field of the development of multi-target strategies for the treatment of Alzheimer’s disease (Figure 4).

When relating the ten most cited articles from the set corresponding to the h-index with the most prolific authors, it is observed that Bolognesi, M. L.; Malawska, B.; and Bartolini, M. account for a significant fraction of the total citations. In particular, the most cited article among these authors, with 547 citations, corresponds to “A perspective on multi-target drug discovery and design for complex diseases,” which provides an integrative view of the molecular and computational foundations underlying the design of multi-target drugs, emphasizing the need for close interaction between medicinal chemistry and clinical research, especially in the context of complex neurodegenerative diseases such as Alzheimer’s disease [8].

The second most cited article, with 288 citations in Web of Science, corresponds to a comprehensive review on the development of multifunctional agents with therapeutic potential against Alzheimer’s disease. This work shows that most of the reported structures correspond to acetylcholinesterase inhibitors, frequently endowed with additional properties that enrich their pharmacological profile. Among the most recurrent functions are the inhibition of β-amyloid aggregation, inhibition of β-secretase and monoamine oxidase, antioxidant and metal-chelating activity, nitric oxide release, as well as interactions with cannabinoid, NMDA, or histamine H_3_ receptors, reinforcing the viability of the multi-target approach for both symptomatic and potentially disease-modifying treatment of Alzheimer’s disease [9].

The third most cited article, with 256 citations, entitled “Multi-Target-Directed Drug Design Strategy: From a Dual Binding Site Acetylcholinesterase Inhibitor to a Trifunctional Compound against Alzheimer’s Disease,” proposes a rational strategy for the evolution of dual-binding-site AChE inhibitors toward trifunctional compounds with promising in vitro profiles against multifactorial syndromes such as Alzheimer’s disease. In this context, the chelation of neurotoxic metals, particularly Fe^3+^ and Cu^2+^, is incorporated as a key element in expanding the therapeutic spectrum of the designed compounds [10].

The fourth most cited article, “Multi-target-directed coumarin derivatives: hAChE and BACE1 inhibitors as potential anti-Alzheimer compounds,” has received 219 citations during the analyzed period. This study addresses the chemical modification of coumarin derivatives through the introduction of halophenylalkylamide functions, achieving compounds with dual-binding-site acetylcholinesterase inhibition and a successful extension of inhibitory activity toward β-secretase (BACE1), thereby consolidating a multifunctional profile relevant for the treatment of Alzheimer’s disease [11].

Finally, the fifth most cited article, “Inhibition of Acetylcholinesterase, β-Amyloid Aggregation, and NMDA Receptors in Alzheimer’s Disease: A Promising Direction for the Multi-Target-Directed Ligands Gold Rush,” accumulates 196 citations in Web of Science. This work focuses on the design of single chemical entities capable of simultaneously modulating multiple key targets, particularly acetylcholinesterase and NMDA receptors, in addition to evaluating the inhibition of AChE-induced β-amyloid aggregation and the antioxidant properties of the compounds, positioning them as relevant candidates within the multi-target ligand paradigm for Alzheimer’s disease [13].

The analysis of the most prolific authors and the articles constituting the core of the highest impact (h-index) reveals that the development of multi-target strategies for Alzheimer’s disease has been driven by a relatively limited group of researchers, whose contributions have been decisive in establishing the conceptual, methodological, and experimental foundations of the field. The convergence between high productivity and high citation impact suggests the existence of consolidated research lines characterized by a coherent evolution from classical medicinal chemistry approaches toward more integrative paradigms that incorporate multiple biological targets and mechanistic perspectives. This pattern reinforces the relevance of the multi-target approach as a mature and continuously expanding strategy within translational Alzheimer’s disease research.

### 2.5. Most Important Journals in the Research on Multi-Target Strategies for the Treatment of Alzheimer’s Disease

The most influential scientific journals in the field of developing multi-target strategies for the potential treatment of Alzheimer’s disease were identified through the application of Bradford’s law, which postulates that a small number of journals concentrate the majority of relevant scientific production on a specific topic, while the remaining articles are distributed across a broader set of peripheral sources. This pattern may be associated with the existence of a highly specialized editorial core, accompanied by progressively wider zones of dispersion.

The analyzed dataset presents an h-index of 71, indicating a high level of impact and scientific maturity in this line of research, together with an exponential annual growth trend, particularly pronounced since 2022. The application of Bradford’s law made it possible to identify a reduced core of high-impact journals responsible for a disproportionate fraction of citations, followed by two peripheral zones grouping a larger number of journals with less frequent contributions.

In this context, 1184 articles related to multi-target strategies were analyzed and classified according to the journals in which they were published. Table 2 summarizes the journals comprising the upper third in terms of productivity, ordered from highest to lowest contribution, which represents the central core of scientific dissemination in this field of research.

In the context of the development of multi-target strategies for the potential treatment of Alzheimer’s disease, Table 2 summarizes the most relevant and influential scientific journals in this field. Among them are European Journal of Medicinal Chemistry (Elsevier), Bioorganic Chemistry (Elsevier), Molecules (MDPI), Bioorganic & Medicinal Chemistry (Elsevier), Bioorganic & Medicinal Chemistry Letters (Elsevier), Journal of Enzyme Inhibition and Medicinal Chemistry (Taylor & Francis), International Journal of Molecular Sciences (MDPI), Journal of Molecular Structure (Elsevier), Frontiers in Pharmacology (Frontiers), and Pharmaceuticals (MDPI).

When grouping this selected set of journals according to their respective publishers, Elsevier accounts for 21.9% of the scientific production, representing more than half of the articles published within the core journals of this research area. This trend may be associated with the interest of authors in disseminating their findings in journals belonging to publishers of high prestige, established trajectory, and rigorous editorial standards, which typically present acceptance rates below 25% [63,64,65]. A similar concentration of publications in Elsevier journals has previously been reported in bibliometric studies related to autism spectrum disorders [66], suggesting a transversal pattern in fields characterized by high biomedical complexity.

Interestingly, the publisher MDPI emerges as the second most prominent publisher with influential journals in the field of multi-target strategies for the treatment of Alzheimer’s disease. This significant concentration of articles may be associated with several factors, including the relatively short peer-review times (an average of 16–18 days) and the brief interval between acceptance and final publication (approximately 4 days) [67,68,69]. However, publication in journals from this publisher requires the payment of Article Processing Charges (APCs), which range between 2700 and 2900 CHF [70]. These costs may be mitigated through institutional agreements within the framework of the Institutional Open Access Program (IOAP) at the global level [71], as well as through discounts associated with special issues by invitation of the editors. In a similar context are the journals Frontiers in Pharmacology and Journal of Enzyme Inhibition and Medicinal Chemistry, which have APCs of 3150 and 3300 CHF, respectively [72,73]. Although hybrid journals offer Open Access options, the costs vary according to the reputation and quartile ranking of the journal, ranging between 4640 and 4030 USD for Q1 journals, and between 3810 and 3150 USD for Q2 journals [65,74,75].

Regarding the prestige and impact of the journals with the highest number of publications, it is observed that seven of the ten belong to the Q1 quartile and are associated with internationally recognized publishers such as Elsevier, Taylor & Francis, MDPI, and Frontiers. The remaining three journals are in the Q2 quartile and are published by Elsevier and MDPI. This preference for journals located in the two highest quartiles may be attributed to their high international visibility, rigorous evaluation processes, and the recognition of quality associated with publishing in these venues, particularly in complex and emerging research niches such as multi-target strategies applied to chronic non-communicable diseases, including Alzheimer’s disease.

From a thematic perspective, it can be observed that eight of the core journals (European Journal of Medicinal Chemistry, Bioorganic Chemistry, Bioorganic & Medicinal Chemistry, Bioorganic & Medicinal Chemistry Letters, Journal of Enzyme Inhibition and Medicinal Chemistry, International Journal of Molecular Sciences, Frontiers in Pharmacology, and Pharmaceuticals) share an explicit interest in biologically relevant molecular interactions, the rational design of bioactive compounds, and the mechanistic understanding of pharmacological effects, although with different levels of methodological requirements. In particular, European Journal of Medicinal Chemistry and Bioorganic Chemistry require a clearly established design rationale and experimental validation of the molecular target [63,64], whereas Journal of Enzyme Inhibition and Medicinal Chemistry focuses specifically on enzymatic inhibition processes and receptor modulation [72]. These journals tend to host more comprehensive and conceptually consolidated studies. In contrast, Bioorganic & Medicinal Chemistry and Bioorganic & Medicinal Chemistry Letters emphasize the publication of new concepts and chemical entities with biological relevance [65,74], with the latter particularly favoring brief communications and initial reports (first reports) of new molecules.

In the case of broader-scope journals such as International Journal of Molecular Sciences, Molecules, and Pharmaceuticals, the provision of exhaustive and reproducible experimental details is required, facilitating the integration of multidisciplinary approaches. Finally, Journal of Molecular Structure is primarily oriented toward the publication of fundamental structural information supported by spectroscopic data, contributing to the molecular understanding of synthesized ligands and their potential application in biological systems [75].

### 2.6. Analysis of Keyword Clusters in the Research on Multi-Target Strategies for the Treatment of Alzheimer’s Disease

The identification and analysis of keywords associated with research on the development of multi-target strategies for the treatment of Alzheimer’s disease during the period 2006–2025 was conducted by applying Zipf’s law, which states that the frequency of occurrence of a term is inversely proportional to its rank within a list ordered by frequency. This approach made it possible to determine the dominant concepts and evaluate the lexical structure of the field of study. In combination with the application of Lotka’s law for the analysis of author productivity and the determination of the h-index to estimate the highest impact core, this procedure allows the characterization of the field from three complementary dimensions: concentration of scientific production, conceptual hierarchy, and academic influence.

**Figure 5:**
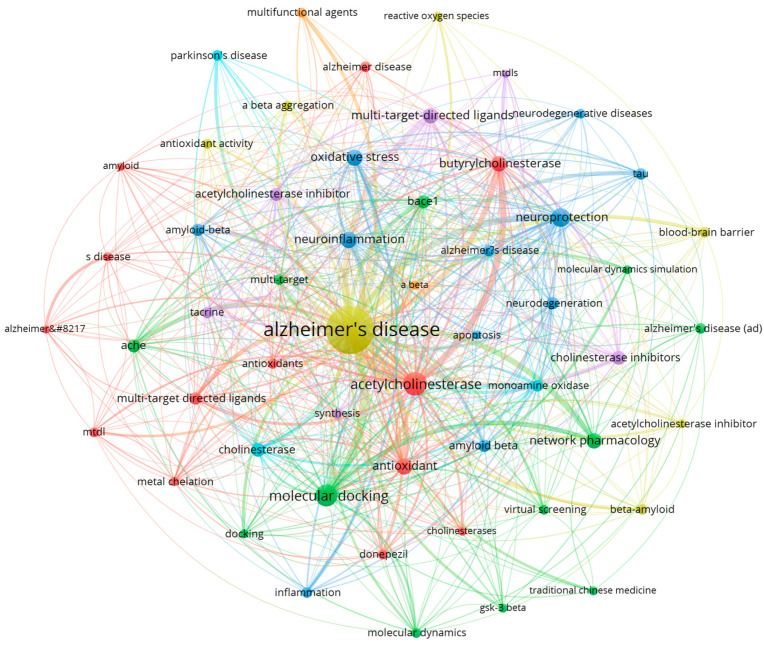
Author keywords and their relations in the development of multitarget strategies for Alzheimer’s disease treatment. The colors correspond to the different clusters grouped by the VOSviewer program.

**Figure 6:**
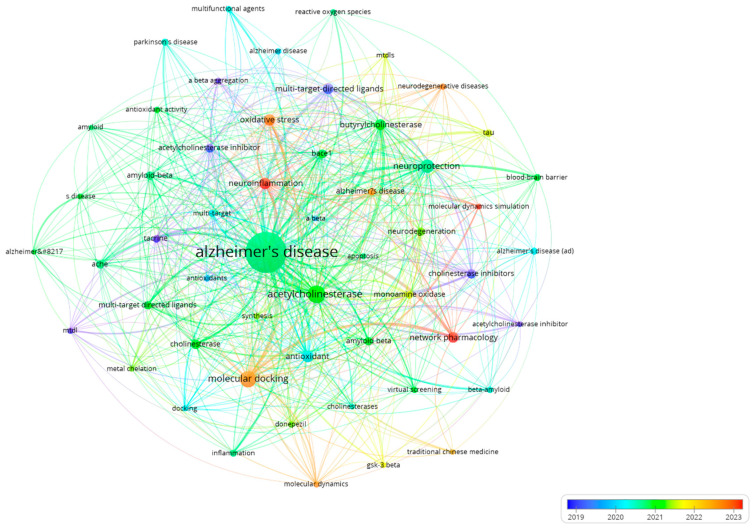
Author keywords with overlaid nodes related to the development of multitarget strategies for Alzheimer’s disease treatment. The colors correspond to the time average determined by the VOSviewer program.

Subsequently, a co-occurrence and clustering analysis was performed using the VOSviewer software to visualize the thematic relationships among the most relevant terms (Figure 5). This procedure enabled the identification of conceptual groupings that could be related to the consolidated research lines within the multi-target paradigm. In addition, a temporal analysis (overlay visualization) (Figure 6) was incorporated, facilitating the distinction between classical terms and emerging concepts and providing an evolutionary perspective on the thematic development of the field.

### 2.7. Keyword Cluster Analysis of Research on Multi-Target Strategies for the Treatment of Alzheimer’s Disease

The keyword co-occurrence visualization allowed the identification of seven thematic clusters structuring the development of multi-target strategies for the treatment of Alzheimer’s disease (Figure 5).

The red cluster is organized around the classical cholinergic axis, with a high presence of terms such as acetylcholinesterase, butyrylcholinesterase, cholinesterases, and donepezil [15,62,76]. This pharmacological core integrates concepts associated with amyloid pathophysiology (amyloid) and complementary mechanisms such as antioxidant and metal chelation [11,13,77,78]. The explicit inclusion of MTDL and multi-target directed ligand confirms the early consolidation of the multi-target paradigm, initially built upon the functional extension of cholinergic inhibitors toward the simultaneous modulation of multiple pathological processes [79,80].

The green cluster is consistent with a transition toward rational design assisted by computational tools. The co-occurrence of targets such as AChE, BACE1, and GSK-3β, together with terms such as molecular docking, molecular dynamic simulation, virtual screening, and network pharmacology, indicates a methodological sophistication aimed at the simultaneous identification of multi-target modulators [81,82,83,84]. The presence of traditional Chinese medicine also suggests the incorporation of natural compounds within virtual screening strategies, expanding the conceptual scope of the field [83].

The blue cluster is structured around central pathophysiological processes, including amyloid beta, apoptosis, neuroinflammation, oxidative stress, and neurodegeneration. Unlike clusters focused on specific enzymatic targets, this grouping emphasizes the cellular and molecular mechanisms underlying disease progression, indicating a growing articulation between pharmacological design and a deeper mechanistic understanding [85,86,87].

The yellow cluster integrates cholinergic inhibition with modulation of the amyloid axis (Aβ aggregation) and control of oxidative stress (reactive oxygen species, antioxidant activity). The inclusion of blood–brain barrier introduces pharmacokinetic considerations, evidencing an evolution toward the design of chemical entities with translational potential and therapeutic feasibility [88,89].

The violet cluster shows a strong orientation toward classical medicinal chemistry, with terms such as synthesis, tacrine, acetylcholinesterase inhibition, and cholinesterase inhibitors. This grouping represents the foundational synthetic–pharmacological core of the multitarget approach, in which structural hybridization becomes established as a central strategy [79,90,91].

The cyan cluster groups cholinesterase, monoamine oxidase, and Parkinson’s disease, suggesting an expansion of the paradigm beyond Alzheimer’s disease. The combined inhibition of cholinesterases and MAO, particularly MAO-B, points to shared mechanisms among neurodegenerative disorders, highlighting a transdiagnostic projection of the multitarget approach [62,92,93].

Finally, the last cluster, composed of the dyad Aβ and multifunctional agents, represents a conceptual synthesis of the field. Rather than a specific technical line, this grouping establishes a direct relationship between the central pathological axis and the therapeutic strategy based on multifunctional agents, suggesting a possible link with the global internalization of the paradigm [94,95,96,97].

### 2.8. Evolutionary Integration and Temporal Analysis of Keywords of Research on Multi-Target Strategies for Treatment of Alzheimer’s Disease

The temporal overlay visualization (Figure 6) supports this structural organization by revealing a progressive evolution of thematic axes. In the initial stages, terms associated with the classical cholinergic paradigm predominate, such as acetylcholinesterase, tacrine, and cholinesterase inhibitors. Subsequently, concepts related to β-amyloid aggregation, metal chelation, and oxidative stress emerge, which may be associated with a functional diversification of pharmacological design.

In more recent years, a shift toward advanced computational tools—molecular docking, molecular dynamics, network pharmacology, and virtual screening—is observed, together with mechanistic targets such as BACE1 and GSK-3β. This transition indicates not only methodological sophistication but also a transformation in the way therapeutic intervention is conceived, moving from predominantly monoenzymatic strategies toward a systemic and integrated approach.

Taken together, the results demonstrate that the multi-target paradigm has evolved from the optimization of cholinergic inhibitors toward a mechanistically grounded therapeutic framework that is methodologically advanced and conceptually aligned with the biological complexity of Alzheimer’s disease.

### 2.9. Scientific Challenges and Future Perspectives in Multitarget Strategies for Alzheimer’s Disease

As evidenced in the previous section, the development of multi-target strategies for the treatment of Alzheimer’s disease has been primarily structured around the cholinergic axis, promoting the design of new chemical entities through computational techniques and their validation predominantly through in vitro studies. Although this trajectory is consistent with a methodological evolution of the paradigm, significant scientific challenges remain.

Importantly, these bibliometric trends should be interpreted as reflecting shifts in scientific attention and design priorities rather than direct evidence of clinical superiority or disease-modifying efficacy. First, despite thematic diversification, the field continues to show a strong dependence on frameworks centered on cholinergic inhibition. Although the extension of acetylcholinesterase inhibitors toward multifunctional ligands represented a significant conceptual advance, the persistence of this axis suggests a certain pharmacological inertia that may limit the exploration of intervention strategies guided by biological networks.

Second, the growing importance of computational methodologies—such as molecular docking, molecular dynamics, virtual screening, and network pharmacology—contrasts with the low bibliometric representation of terms associated with clinical validation, biomarker integration, or patient stratification. This asymmetry highlights a persistent translational bottleneck, in which advances in rational design are not fully matched by systematic in vivo validation and development oriented towards clinical practice.

Third, although mechanisms such as neuroinflammation, oxidative stress, and apoptosis have gained prominence, the systemic complexity of Alzheimer’s disease remains only partially integrated into multi-target design. However, synaptic dysfunction, tau-mediated toxicity, neuronal network failure, and plasticity impairment (key determinants of AD progression) remain comparatively underrepresented in the keyword landscape, underscoring the need for broader mechanistic integration. Future efforts should move beyond additive approaches toward network-based strategies that prioritize key regulatory nodes within interconnected pathological cascades.

Finally, the emergence of transdiagnostic elements, including associations with Parkinson’s disease, opens an opportunity to expand the multi-target framework toward mechanisms shared among neurodegenerative disorders. In this context, the incorporation of artificial intelligence-assisted design, systems pharmacology, and the integration of multi-omics data could define the next evolutionary stage of the field. Taken together, these perspectives point toward a transition from structurally multifunctional compounds to therapeutic design oriented toward mechanistically grounded biological networks.

Thus, the field appears to be moving from target accumulation toward mechanistically prioritized network modulation, but true translational success will depend on whether these conceptual advances can be validated in vivo and ultimately in clinical settings.

## 3. Materials and Methods

### 3.1. General Methodology

The dataset used in this study was retrieved from the Web of Science Core Collection on 13 January 2026. The search included the following indexes: Science Citation Index Expanded (SCI-Expanded), Social Sciences Citation Index (SSCI), Arts & Humanities Citation Index (AHCI), Conference Proceedings Citation Index-Science (CPCI-S), Conference Proceedings Citation Index-Social Science & Humanities (CPCI-SSH), Book Citation Index-Science (BKCI-S), Book Citation Index-Social Science & Humanities (BKCI-SSH), and Emerging Sources Citation Index (ESCI).

The literature search was conducted using the thematic query field (TS), which simultaneously scans the title, abstract, author keywords, and Keywords Plus^®^ [98]. Additional filtering criteria were applied to refine the dataset, restricting the document type to research articles.

Following the methodological framework proposed in the Guidelines for advancing theory and practice through bibliometric research [99], the analysis combined performance analysis and science mapping approaches. The performance analysis was conducted using classical bibliometric indicators, including the laws of Price’s Law [100,101], Lotka’s Law [46,47], Bradford’s Law [102,103], and the Hirsch Index (h-index) [104,105]. In parallel, science mapping techniques were applied to explore collaboration patterns through co-authorship networks using the software VOSviewer (version 1.6.20; Centre for Science and Technology Studies, Leiden University). Further methodological details are provided in Table 3.

The 2006–2025 period was selected because it represents the first interval with continuous and sustained scientific production, allowing a reliable application of bibliometric growth models.

### 3.2. Eligibility Criteria

Inclusion criteria comprised peer-reviewed research articles indexed in the Web of Science Core Collection, written in English, and explicitly addressing dual- or multi-target strategies in Alzheimer’s disease.

Exclusion criteria included reviews, conference abstracts, editorials, and studies not directly related to multitarget pharmacological design.

### 3.3. Debugging Pipeline

The initial dataset (*n* = 1685) was refined by restricting document type to articles (*n* = 1205), followed by a temporal filter (2006–2025), yielding a final corpus of 1184 records.

### 3.4. Thresholds in VOSviewer

Threshold selection in VOSviewer analyses was performed to reduce network noise, improve visualization interpretability, and retain the most representative elements within each bibliometric unit. Different thresholds were established according to the characteristics and distribution patterns of the analyzed variables, following commonly applied criteria in bibliometric and science-mapping studies.

For co-authorship analysis, a minimum of 7 documents per author and 10 documents per institution were required. Countries were filtered using a threshold of 45 documents to focus on the most productive contributors at the global level.

For keyword co-occurrence analysis, the threshold was estimated according to the square root of the total keywords (SQRT (2892) = 53.8), resulting in 54 keywords with a minimum occurrence threshold of 12. This criterion was applied to reduce noise while preserving the most representative and thematically relevant terms, consistent with the Zipfian distribution of keyword frequencies.

For journal analysis, a threshold of 12 documents per source was used, consistent with Bradford’s law, to identify the core journals in the field.

## 4. Conclusions

The present bibliometric study performed a characterization of the structural, thematic, and temporal evolution of the development of multi-target strategies for the treatment of Alzheimer’s disease during the period 2006–2025. The analysis of productivity, impact, and keyword co-occurrence indicates that the MTDL paradigm has progressively consolidated as a central framework in medicinal chemistry oriented toward neurodegenerative diseases.

Scientific productivity in this field shows a significant concentration in China, a phenomenon that appears to be primarily determined by the structural capacity of its national science and technology systems, access to competitive funding, and integration into international collaboration networks, rather than by the epidemiological burden of the disease considered in isolation. In this context, institutions such as Sun Yat-sen University, China Pharmaceutical University, Nanyang Normal University, and the Chinese Academy of Sciences stand out, together with highly prolific authors such as Zhipei Sang (Nanyang Normal University), Ondrej Soukup (University Hospital Hradec Králové), Manuela Bartolini (University of Bologna), and Jan Korabecny (University of Defense).

In its early stages, the field was structured around the classical cholinergic axis, particularly the inhibition of acetyl- and butyrylcholinesterase as the predominant strategy. Subsequently, a functional diversification is observed toward the integration of complementary mechanisms, including β-amyloid aggregation, oxidative stress, and metal chelation. More recently, the incorporation of advanced computational tools and approaches based on network pharmacology could be related to a methodological sophistication aligned with an increasingly systemic understanding of neurodegenerative pathology.

Nevertheless, the results also highlight persistent challenges, especially regarding the structural dependence on cholinergic platforms and the limited representation of clinical validation and biomarkers in the analyzed literature. These findings suggest that the next evolutionary stage of the field will require a transition from predominantly structural approaches toward strategies guided by biological networks, multi-omics integration, and robust translational validation.

Taken together, this study not only describes the historical trajectory of the multi-target paradigm in Alzheimer’s disease but also provides an interpretative framework to understand its conceptual maturation and to guide future research directions in the rational design of therapeutic agents for complex neurodegenerative disorders. In this context, the observed evolution should be interpreted as a bibliometrically inferred conceptual transition, not as direct evidence of therapeutic efficacy.

## Figures and Tables

**Table 1:** Institutions around the world contributing most to research related to the development of multi-target compounds for Alzheimer’s disease.

Entry	Institution	Country	Percentage of Contribution
1	Sun Yat Sen University	China	0.8
2	China Pharmaceutical University	China	0.7
3	Nanyang Normal University	China	0.7
4	Università degli Studi di Bari Aldo Moro	Italy	0.7
5	Chinese Academy of Sciences	China	0.7
6	Consejo Superior de Investigaciones Científicas (CSIC)	Spain	0.7
7	University Hospital Hradec Králové	Czech Republic	0.6
8	University Lisbon	Portugal	0.6
9	Jagiellonian University	Poland	0.6
10	University of Coimbra	Portugal	0.6
		Total	6.7

**Table 2:** Journals from the research nucleus in the multi-target strategy for treatment of Alzheimer’s disease.

Entry	Journal	Publisher	Impact Factor 2024	Category ^a^	Per. ^b^	P.T. ^c^
1	European Journal of Medicinal Chemistry	Elsevier France-Editions Scientifiques Medicales Elsevier (Paris, France)	5.9	Chemistry, Medicinal (Q1)	8.7	Hy
2	Bioorganic Chemistry	Academic Press Inc Elsevier Science (San Diego, CA. USA)	5.9	Biochemistry & Molecular Biology (Q1) Chemistry, Organic (Q1)	5.3	Hy
3	Molecules	MDPI (Basel, Switzerland)	4.6	Chemistry, Multidisciplinary (Q2) Biochemistry & Molecular Biology (Q2)	4.0	OA
4	Bioorganic & Medicinal Chemistry	Pergamon-Elsevier Science Ltd. (Oxford, UK)	3.0	Chemistry, Medicinal (Q3) Biochemistry & Molecular Biology (Q3) Chemistry, Organic (Q1)	3.7	Hy
5	Bioorganic & Medicinal Chemistry Letters	Pergamon-Elsevier Science Ltd. (Oxford, UK)	2.2	Chemistry, Medicinal (Q3) Chemistry, Organic (Q2)	2.4	Hy
6	Journal of Enzyme Inhibition and Medicinal Chemistry	Taylor & Francis Ltd. (Abingdon, UK)	5.4	Chemistry, Medicinal (Q1) Biochemistry & Molecular Biology (Q1)	2.2	OA
7	International Journal of Molecular Sciences	MDPI (Basel, Switzerland)	4.9	Chemistry, Multidisciplinary (Q1) Biochemistry & Molecular Biology (Q2)	2.0	OA
8	Journal of Molecular Structure	Elsevier (Amsterdam, The Netherlands)	4.7	Chemistry, Physical (Q2)	1.8	Hy
9	Frontiers in Pharmacology	Frontiers Media SA (Switzerland)	4.8	Pharmacology & Pharmacy (Q1)	1.6	OA
10	Pharmaceuticals	MDPI (Basel, Switzerland)	4.8	Pharmacology & Pharmacy (Q1) Chemistry, Medicinal(Q1)	1.5	OA
				Total	33.2	

^a^ Category = Category reported by Web of Science, and in parentheses, the quartile reported by Web of Science, according to 2024 statistics. ^b^ Per = Percentage according to the total number of articles analyzed (1184 published articles). ^c^ P.T. = Publication type: Subscription (S), Hybrid (Hy), or Open Access (OA).

**Table 3:** Characterization of the document corpus to be analyzed.

Variable	Value (or Sample, *n*)	Unit	Subsampling Criterion
Documents	1184	Article	Hirsch’s index (h-index)
Time	2006–2025	Years	Period without blanks, Price’s Law ^(1)^
Place (Affiliation)	78	Country/Territory	Census
Authors	6833	Person	Lotka’s Law
Keywords and Keywords Plus	2892 2307	Words	Zipf’s Law
Journals	351	Journal	Bradford’s Law

^(1)^ Price’s laws allow us to examine the exponential growth of science, measured.

## Data Availability

The original data presented in the study are openly available in the Web of Science Core Collection. The datasets generated and analyzed during the current study, including bibliometric indicators, co-authorship networks, and keyword co-occurrence matrices generated using VOSviewer, are available in the Supplementary Materials (ZIP file).

## Supplementary Materials

The following supporting information can be downloaded at: https://www.mdpi.com/article/10.3390/ph19071024/s1, Figure S1: Most prolific institutions and their collaboration networks to the study of multi-target compounds as a therapeutic strategy for the treatment of Alzheimer’s disease; Dataset S1: database.zip (raw and processed bibliometric data retrieved from the Web of Science Core Collection, including data used for co-authorship and keyword co-occurrence analyses).
