# Supplementary material for: Evolution of Multitarget Strategies for Alzheimer’s Disease: From Cholinergic Inhibition to Network-Oriented Therapeutic Design (2006–2025)
Source: Pharmaceuticals (Basel). 2026 Jun 30;19(7):1024. doi: 10.3390/ph19071024 (PMC13415210; doi:10.3390/ph19071024)
Supplement: Supplementary file 1 [file pharmaceuticals-19-01024-s001.zip › Pharmaceuticals_Supplementary-Materials.pdf]

pharmaceuticals

SUPPLEMENTARY MATERIALS

Article

Evolution of Multitarget Strategies for Alzheimer's Disease: From Cholinergic Inhibition to Network-Oriented Therapeutic Design (2006–2025)

Jaime Mella[1,2], Alejandro Vega-Muñoz[3,4], Mauricio Soto[5], Daniel Moraga[6], Javier Campanini-Salinas[7], Eduardo Sandobal-Obando[8], Nicolás Contreras-Barraza[9], Guido Salazar-Sepúlveda[10,11], Natalia Salas-Guzmán[12], Remik Carabantes-Silva[13], Marco Mellado[14,15]*

[1] Instituto de Química, Facultad de Ciencias, Universidad de Valparaíso, Valparaíso 2360102, Chile; jaime.mella@uv.cl
[2] Centro de Investigación, Desarrollo e Innovación de Productos Bioactivos (CInBIO), Universidad de Valparaíso, Valparaíso 2360102, Chile.
[3] Laboratorio de Bienestar y Comportamiento Organizacional, Universidad Central de Chile, Santiago 8330507, Chile; alejandro.vega@ucentral.cl
[4] Facultad de Ciencias Empresariales, Universidad Arturo Prat, Santiago 8340232, Chile.
[5] Departamento de Química, Universidad Técnica Federico Santa María, Av. España 1680, Valparaíso 2340000, Chile; mauricio.sotoc@usm.cl
[6] Laboratorio de Fisiología, Departamento de Ciencias Biomédicas, Facultad de Medicina, Universidad de Tarapacá, Arica 1000000, Chile; dmoraga@academicos.uta.cl
[7] Escuela de Química y Farmacia, Facultad de Ciencias, Universidad San Sebastián, Lago Panguipulli 1390, Puerto Montt 5501842, Chile; javier.campanini@uss.cl
[8] Escuela de Psicologia, Facultad de Ciencias Sociales y Humanidades, Universidad Autónoma de Chile, Temuco 4800916, Chile. mail: eduardo.sandoval@uautonoma.cl
[9] Facultad de Ciencias Económicas y Administrativas, Pontifi cia Universidad Católica de Valparaíso, Valparaíso 2340025, Chile; nicolas.contreras@pucv.cl
[10] Facultad de Ingeniería, Universidad Católica de la Santísima Concepción, Concepción 4090541, Chile; gsalazar@ucsc.cl
[11] Facultad de Ingeniería y Negocios, Universidad de Las Américas, Concepción 4090940, Chile.
[12] Facultad de Educación y Ciencias Sociales, Universidad Finis Terrae, Santiago, Chile; nsalas@uft.cl
[13] Facultad de Ciencias Naturales, Matemáticas y del Medio Ambiente, universidad Tecnológica Metropolitana, Santiago 8330383, Chile; r.carabantes@utem.cl
[14] Dirección de Investigación, Universidad Bernardo O'Higgins, Santiago 8370993, Chile.
[15] Laboratorio de Espectroscopía y Química Aplicada, Grupo de Investigación en Ciencias Biomédicas Aplicadas, Universidad Central de Chile, Santiago 8330546, Chile; marco.mellado@ucentral.cl
* Correspondence: Dr. Marco Mellado, Email: marco.mellado@psh.ubo.cl

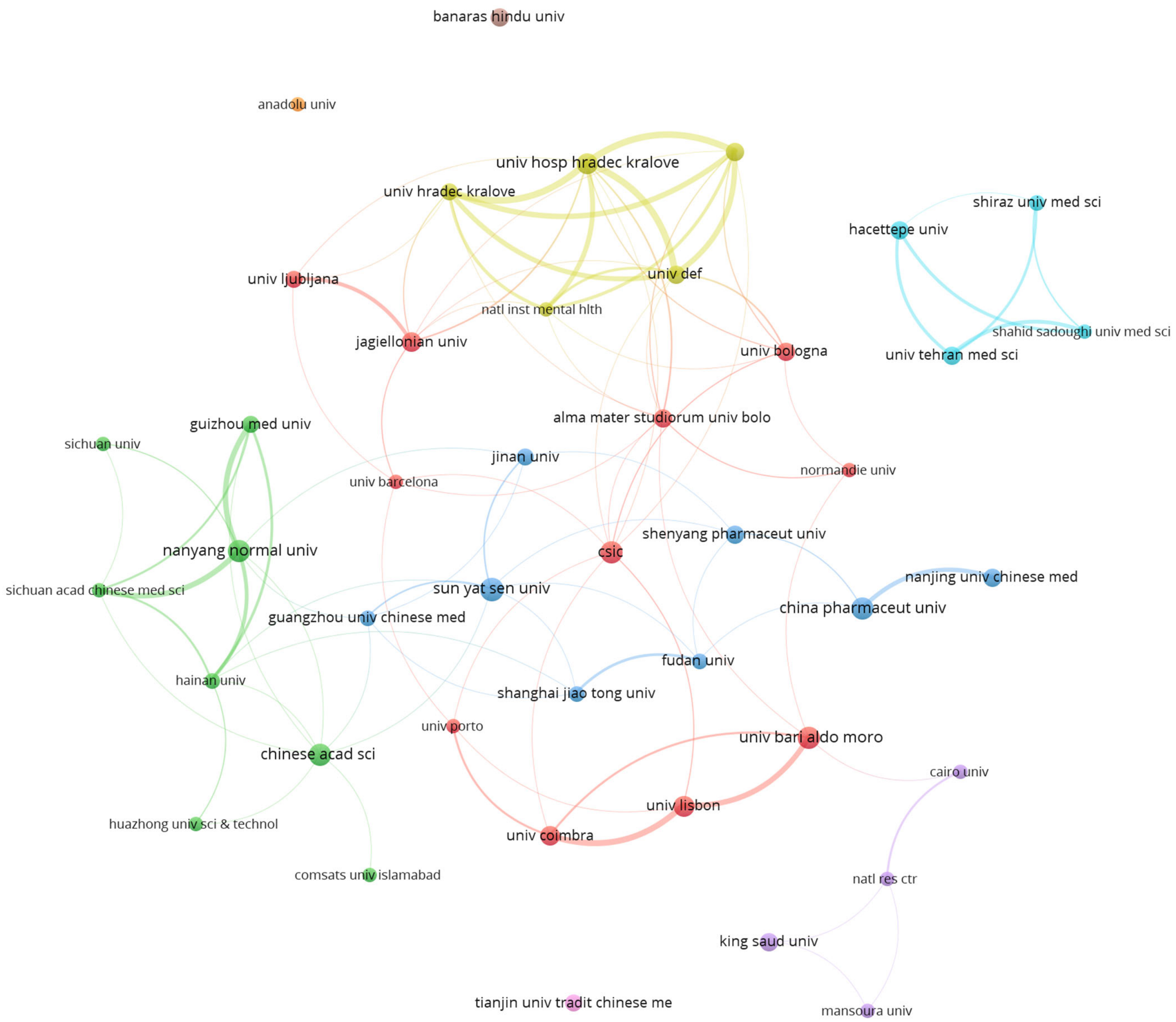

Figure S1: Most prolific institutions and their collaboration networks to the study of multi-target compounds as a therapeutic strategy for the treatment of Alzheimer's disease.
